# The Effectiveness of Autologous Platelet-Rich Plasma Topical Application Versus Injection in the Healing of Chronic Venous Ulcers

**DOI:** 10.7759/cureus.117003

**Published:** 2026-09-27

**Authors:** Neeraja Neena Nair, Thrishuli PB

**Affiliations:** 1 General Surgery, JSS Academy of Higher Education and Research, Mysuru, IND

**Keywords:** chronic venous insufficiency (cvi), chronic venous ulcers, lipodermatosclerosis, platelet-rich plasma, varicose veins

## Abstract

Introduction

Chronic venous leg ulcers (VLUs) are common lower-limb wounds associated with delayed healing, recurrence, pain, impaired mobility, reduced quality of life, and increased healthcare costs. Although compression therapy remains the standard treatment, many ulcers heal slowly or remain resistant, highlighting the need for effective adjunctive therapies.

Autologous platelet-rich plasma (PRP) is rich in platelets and growth factors that promote angiogenesis, fibroblast proliferation, collagen synthesis, granulation tissue formation, and re-epithelialization, making it a promising regenerative therapy for chronic venous ulcers. However, existing evidence is limited due to small sample sizes, methodological heterogeneity, and variability in PRP preparation. Systematic reviews have therefore recommended well-designed prospective randomized trials to establish its efficacy and safety.

Aims and objectives

The study aims to evaluate the clinical efficacy of PRP topical application, PRP injection, and saline dressings in chronic VLUs, with the primary objective based on complete ulcer healing and percentage reduction in ulcer size, while assessing ulcer recurrence and early treatment-related complications constituted the secondary endpoints. Recurrence here is defined as re-appearance of the ulcer after its complete healing at the same site.

Methodology

This prospective randomized controlled trial was conducted over 18 months at JSS Medical College and Hospital, Mysuru, Karnataka, India. A total of 63 adults with chronic VLUs (>6 weeks) were randomly allocated into three groups: group A (topical PRP dressing), group B (local PRP injection), and group C (saline dressing). All patients received a standard four-layer compression therapy. Autologous PRP was prepared using a double-spin centrifugation technique and administered weekly for four weeks. Patients were followed for six months. Primary outcomes included complete ulcer healing and percentage reduction in ulcer area, while recurrence and treatment-related complications were assessed as secondary outcomes using SPSS Version 28 (p < 0.05).

Results

Baseline comparability was noted among groups for age, sex, BMI, HbA1c, platelet count, ankle-brachial pressure index, ulcer duration, and initial ulcer area. At six weeks, complete healing of the ulcer was much greater in group B (33.3%) than in group A (9.5%) and group C (0%) (p = 0.0064). At three months, the mean ulcer area was minimal in group B (1.30 ± 1.61 cm²) compared to group A (3.15 ± 3.01 cm²) and group C (6.19 ± 4.49 cm²) (p < 0.0001). The percentage reduction in ulcer area at three months was highest in group B (91.80 ± 8.05%), followed by group A (78.29 ± 17.68%) and group C (62.34 ± 19.06%). The mean healing duration was the shortest in group B (11.20 ± 2.18 weeks) (p = 0.0001). The rates of recurrence and complications were minimal and similar.

Conclusion

Autologous PRP is a safe and effective adjunct for chronic VLUs. Both PRP topical application and injection improved healing compared with saline dressing, while PRP injection achieved earlier healing, greater ulcer area reduction, and shorter healing time, making it the preferred PRP delivery method.

## Introduction

Chronic venous leg ulcers (CVLUs) are among the most common chronic lower-limb wounds and constitute a major clinical and public health problem because of delayed healing, frequent recurrence, pain, impaired mobility, reduced quality of life, and high healthcare costs. CVLU is a prevalent type of chronic lower-limb ulceration, signifying the last stage of persistent venous hypertension resulting from chronic venous illness, venous reflux, venous blockage, or post-thrombotic syndrome. Venous leg ulcers (VLUs) are clinically significant due to their painful nature, protracted healing process, high recurrence rate, and association with extended disability, decreased mobility, sleep disturbances, social isolation, and substantial healthcare costs. A recent systematic review and meta-analysis indicated a pooled prevalence of 0.32% and a pooled incidence of 0.17%, highlighting significant variability among studies; this elucidates why VLUs continue to pose a substantial public health challenge despite their ubiquity in routine practice [1-3].

Compression therapy remains the standard treatment; however, many ulcers fail to heal or require prolonged treatment, highlighting the need for adjunctive therapies that can accelerate healing and reduce chronicity [4,5]. The standard treatment for VLUs relies on precise diagnosis, rectification of venous pathology where applicable, debridement and preparation of the wound bed, infection control, decrease of edema, and, primarily, compression therapy. Primary pathology in venous ulcers is the chronic venous hypertension that occurs due to reflux in superficial venous, perforator and/or deep venous systems, or deep venous/pelvic obstruction; thus, unless the chronic venous hypertension has been completely addressed in the chosen treatment modality, CVLU cannot attain complete healing [6,7].

The aim in management of chronic VLU is to eliminate/reduce the source of pathological venous hypertension and thereby promote ulcer healing and prevent recurrence. This involves the identification of the exact venous pathology using venous duplex ultrasound, followed by specific surgery for the same. These surgeries include endovenous thermal ablation such as EVLA (endovenous laser ablation) or RFA (radiofrequency ablation); non-thermal ablation such as foam sclerotherapy; or conventional surgery for the superficial venous incompetence and reflux. Incompetent perforators, particularly those associated with the ulcer bed, may be treated selectively with endoscopic/open techniques. Deep venous reflux alone does not preclude treatment of superficial reflux, while significant iliac or deep venous obstruction may require endovascular recanalization, angioplasty, and stenting. Current recommendations, notably the 2024 German S2k guideline, prioritize compression in the therapy of VLUs and characterize adjunctive therapies as supplementary rather than substitutive to effective VLU care. The principal inquiry in the platelet-rich plasma (PRP) literature is not whether PRP should supplant compression but whether autologous PRP injection or topical treatment might expedite healing when utilized in conjunction with normal therapy for chronic, hard-to-heal ulcers [6-8].

Autologous PRP has emerged as a promising regenerative therapy [9] because it contains a high concentration of platelets and growth factors that promote angiogenesis, fibroblast proliferation, granulation tissue formation, and re-epithelialization. In chronic venous ulcers, where healing is compromised by persistent venous hypertension, inflammation, and tissue hypoxia, PRP may help restore the local wound-healing environment, providing a strong biological rationale for its use as an adjunct to conventional treatment [10]. Non-healing VLUs are linked to a pro-inflammatory cytokine environment, with little data indicating increased levels of IL-1α, IL-6, IL-8, TNF-α, and VEGF in non-healing wounds, although TGF-β1 often increases throughout the healing process. This indicates that chronic VLUs are not only poorly healing wounds but rather dysregulated wounds, and PRP may assist by modulating inflammation while concurrently providing pro-healing mediators [11].

Although previous studies and pooled evidence suggest that PRP may improve healing rates and reduce ulcer size compared with standard care, the evidence remains limited due to small sample sizes, methodological heterogeneity, and inadequately powered randomized trials [9,12,13]. Therefore, the true therapeutic benefit of PRP remains uncertain, highlighting the need for well-designed prospective randomized studies and supporting the rationale for the present study.

## Materials and methods

This prospective, randomized controlled trial was conducted over 18 months, from March 2024 to August 2025, in the Department of General Surgery at JSS Medical College and Hospital, Mysuru, Karnataka, India, with support from the Department of Laboratory Pathology for laboratory investigations and PRP processing. After attaining clearance from the Institutional Ethics Committee (IEC), the trial was registered under Clinical Trials Registry-India, and each case was entered into the registry subsequently. The study included adult patients attending the General Surgery outpatient department with CVLUs of more than six weeks' duration who fulfilled the predefined eligibility criteria. Eligible participants were randomly assigned to the treatment groups using the sealed envelope method. A single-blind design was employed, and no crossover between groups was permitted during the study period.

The sample size was calculated based on an anticipated 50% difference in healing rates between the treatment groups, as reported by Elbarbary et al. [14]. Assuming a study power of 80%, a 95% confidence level, and a significance level (α) of 5%, the minimum required sample size was estimated to be 19 participants per group. After allowing for a 10% dropout rate, the final sample size was increased to 21 participants in each group.

Randomization and blinding

Eligible participants were randomized using the sealed-envelope method, in which three identical sealed envelopes representing the three treatment modalities were used to ensure an equal probability of allocation to each study group. Crossovers between groups were not permitted. A single-blind design was maintained, with participants of a particular group remaining unaware of the treatment modality applied to the other two groups and with the outcome assessor remaining unaware of treatment allocation. Patients were randomly allocated into three groups: group A received topical PRP dressing; group B received local PRP injections into the ulcer floor, edges, and depth; and group C received saline dressing as the control. In addition to the allocated intervention, all participants received standard compression therapy, the current gold standard treatment for CVLUs, throughout the study period.

Inclusion criteria

Adult patients with CVLUs with insufficient healing for a minimum of six weeks were enrolled in the trial. Patients were required to possess an ankle-brachial pressure index (ABPI) of no less than 0.9 and a platelet count ranging from 150,000 to 450,000/mm³.

Exclusion criteria

Patients were excluded for having renal, hepatic, or cardiac failure; uncontrolled diabetes mellitus (HbA1c >12); ulcers resulting from electrical, chemical, or radiation burns; malignancy; connective tissue disorders; untreated underlying osteomyelitis; pregnancy; lactation; local active infection; or receiving antiplatelet agents, steroids, or immunosuppressive medications. Patients who were hesitant to provide informed consent or had a history of inadequate adherence to compression treatment were also excluded. Furthermore, individuals who had underwent or required endovenous surgery, great saphenous vein (GSV) stripping, or sclerotherapy during the trial period were excluded from the study.

Patient assessment and baseline investigations

A detailed history was obtained from all participants, followed by general and local examinations, to confirm the diagnosis of a pure VLU and assess eligibility. Demographic and clinical parameters, including age, sex, smoking status, diabetes mellitus, and body mass index (BMI), were recorded. Local examination included the assessment of peripheral pulses, ABPI, and ulcer characteristics. Venous duplex imaging was performed to identify venous reflux or obstruction contributing to venous hypertension. Baseline investigations included complete blood count, glycosylated hemoglobin, fasting and postprandial blood sugar levels, renal and liver function tests, routine urine examination, wound culture and sensitivity, and venous Doppler of the affected limb.

Ulcer assessment and measurement

Ulcers were assessed for duration, laterality, edge, margin, floor characteristics, and requirement for debridement or antibiotics. Previous episodes of complete healing and duration before recurrence were documented. Ulcer surface area was measured using the ulcer tracing method, wherein the ulcer outline was transferred onto graph paper with 1-cm² markings for area calculation. In patients with multiple ulcers, the chronic ulcer persisting for more than six weeks and of adequate size was selected as the target ulcer. A CONSORT (CONsolidated Standards Of Reporting Trials) diagram regarding the major events in the study is shown in Figure 1.

**Figure 1:**
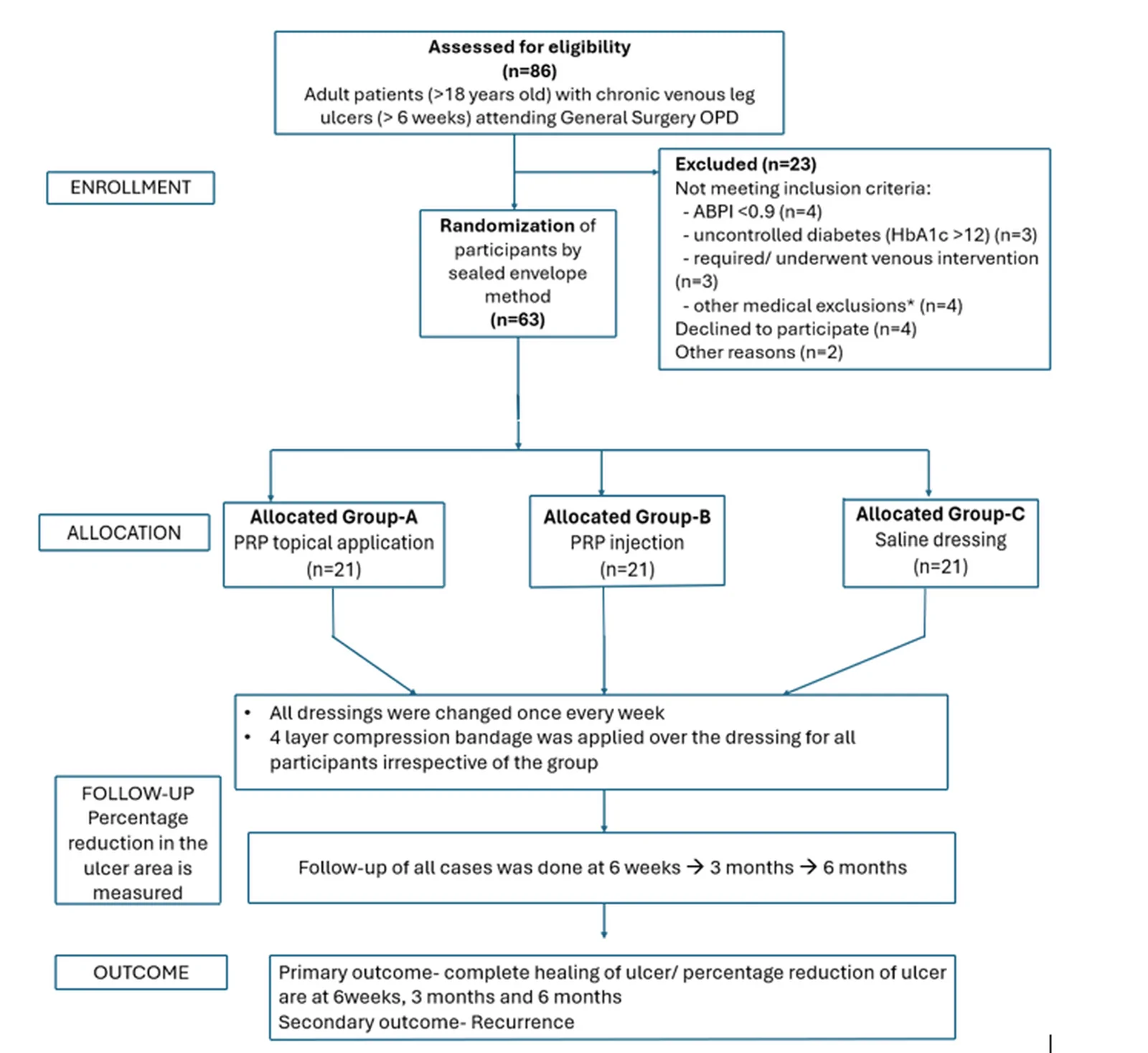
CONSORT flow diagram of participant enrolment, randomization, allocation, follow-up, and outcomes in the study *Renal, hepatic, or cardiac failure; malignancy; connective tissue disorders; untreated underlying osteomyelitis; local active infection; or receiving antiplatelet agents, steroids, or immunosuppressive medications. ABPI, ankle-brachial pressure index; CONSORT, CONsolidated Standards Of Reporting Trials; OPD, outpatient department; PRP, platelet-rich plasma

For all patients, after cleaning the ulcer with normal saline, two swabs were taken from clean, viable areas of the ulcer bed and sent for antibiotic culture and sensitivity analysis. Patients were initially started with empirical antibiotics for the ulcer, later converting them to systemic antibiotics specific to the culture sensitivity report. Before initiating study therapy, ulcers with evidence of infection, necrotic tissue, or positive wound cultures underwent debridement. With every change in PRP dressings (once a week) for four weeks, antibiotic culture sensitivity was performed, and antibiotics were changed accordingly. A negative culture sensitivity report was not needed before the initiation of PRP dressings and during the course of the study; if a negative antimicrobial assessment was attained, antibiotics were discontinued. During the course of follow-up for a total of six months, if the ulcer was reducing in size but still persistent and associated with pain, edema, pus discharge, culture sensitivity analysis would be repeated and antibiotics restarted as indicated.

Preparation of platelet-rich plasma

Autologous venous blood (volume depending on ulcer size) was collected under aseptic conditions and mixed with 5 mL of 3.8% sodium citrate. On double-spin centrifugation technique, 10-20 mL of whole blood yields approximately 1 mL of PRP, and in this study protocol, 1 mL of PRP was to be applied (topical application/ injection) for 1 cm² of ulcer area, with a maximum of 10 mL of PRP per session. A desktop double-spin centrifugation system (MTH-0652A, Micro Teknik, Ambala, Haryana, India) was used in this study. It has a maximum speed of 4,000 rpm with a maximum RCF (relative centrifugal force) of 2600 x g and a rotor capacity of 4 x 20 mL. The final PRP characteristics after two centrifuges was considered to adhere to the hospital protocol, i.e., to have a platelet enrichment factor of 3-5 x baseline whole blood platelet concentration (~1-1.5x10⁶/µL) with minimal to negligible RBC contamination and leukocyte-poor PRP. In the double-spin technique, whole blood undergoes a first low-speed “soft spin” (100-300 × g for 5-10 min) to separate erythrocytes from platelet-containing plasma. The plasma is transferred and subjected to a second higher-speed “hard spin” (400-700 × g for 10-17 min), concentrating platelets into a pellet. Most platelet-poor plasma is discarded, and the pellet is resuspended to obtain PRP.

Intervention protocol and follow-up

Patients were assessed weekly for six weeks, followed by assessment at three months and six months. Before each session, PRP was freshly prepared, and an 18-gauge needle was used to extract and inject the PRP from centrifuged collection tube. Group A received topical PRP dressing, where PRP was topically applied as 1 mL of PRP per 1cm² and then covered with normal saline-soaked sterile gauze. Group B received PRP injections predominantly into the edges than the floor of the ulcer, at a depth of 5 mm and following the protocol of 1 mL of PRP per 1 cm², after which normal saline-soaked sterile gauze was used to cover the ulcer to maintain a moist wound environment. Group C received normal saline dressing alone. All ulcers were covered with a layer of absorbent sterile gauze dressings followed by a layer of cotton pad and roller bandage, on top of which a single elastic compression bandage was applied from the base of the toes to just below the knee joint. In groups A and B, PRP therapy was administered weekly once for up to four weeks, following which saline dressings were continued until complete healing of the ulcer. Based on the degree of soakage of dressing in the PRP therapy groups, only the exterior of the dressings was changed and reinforced, with the inner gauze layer kept intact if increased visible soakage was noted.

Four-layer compression therapy was performed for all patients irrespective of which group they belonged to until complete healing of the ulcer, followed by class II compression stockings for continuation. Four-layer compression bandages were also changed once every week, with earlier change needed only if the bandage became wet, soiled, loose, displaced, or saturated with exudate. Patients were followed up at monthly intervals for six months after completion of the intervention.

Outcome measures and statistical analysis

The primary outcomes were complete ulcer healing and percentage reduction in ulcer area at six weeks, three months, and six months. Secondary outcomes included ulcer recurrence and treatment-related adverse effects such as irritation, erythema, pain, and discomfort. Data were analyzed using SPSS Version 28 (IBM Corp., Armonk, NY). Continuous variables were expressed as mean and standard deviation, while categorical variables were presented as frequencies and percentages. One-way ANOVA with Tukey’s post hoc analysis and repeated-measures ANOVA with Bonferroni correction were used for statistical comparisons. A p-value of <0.05 was considered statistically significant.

## Results

A total of 63 patients were included in the study, with 21 patients each in the groups: PRP application (group A), PRP injection (group B), and saline dressing (group C). The baseline characteristics, including age, sex, BMI, HbA1c, platelet count, ABPI, and ulcer characteristics, were comparable among the three groups, with no statistically significant differences observed. The age distribution across the three groups is presented in Table 1.

**Table 1:** Distribution of patients according to age group across the three study groups One-way ANOVA (mean age): F = 1.069, p = 0.3497 ANOVA, analysis of variance; PRP, platelet-rich plasma; SD, standard deviation

Age Group	Group A (PRP Application) (n=21)	Group B (PRP Injection) (n=21)	Group C (Saline Dressing) (n=21)	Total (n=63)
<30 years	3	3	1	7
30–39 years	2	5	5	12
40–49 years	4	4	5	13
50–59 years	6	7	7	20
≥60 years	6	2	3	11
Mean ± SD (years)	48.43 ± 14.41	42.90 ± 12.36	46.76 ± 10.61	46.03 ± 12.57

The majority of patients in all three groups fell in the age group of 50-59 years and were overweight or obese. Out of all the comorbidities noted, smoking was more prevalent in group A (13 patients) compared to groups B and C (5 and 6 patients, respectively), with a statistically significant difference (p = 0.0216). A previous history of deep vein thrombosis (DVT) was significantly different across groups (p = 0.0349), with group B having the highest frequency (12 patients). The distribution of comorbidities and risk factors is shown in Table 2.

**Table 2:** Distribution of comorbidities and risk factors across the three study groups Pearson chi-square test was used; p < 0.05 was considered statistically significant. *Smoking can be associated with delayed healing in venous leg ulcers. **Pevious DVT is taken as the post- thrombotic secondary cause of varicose veins. DVT, deep vein thrombosis; PRP, platelet-rich plasma

Variable	Group A (PRP Application) (n=21)	Group B (PRP Injection) (n=21)	Group C (Saline Dressing) (n=21)	χ² value	p-Value
Smoking, n (%) (yes)	13	5	6	7.673	0.0216*
Diabetes mellitus, n (%) (yes)	2	0	2	2.136	0.3438
Previous DVT, n (%) (yes)	4	12	7	6.711	0.0349**

Most ulcers were single, were medially located, and had similar baseline duration and surface area across groups. Debridement was performed in 11, 10, and 3 patients in groups A, B, and C, respectively. The difference in debridement rates across groups was statistically significant (p = 0.0216), with group C having a notably lower frequency. Culture positivity and reflux type distribution were not significantly different across groups (p = 0.2389 and p = 0.2767, respectively). GSV reflux was the predominant finding in all three groups. The detailed baseline ulcer characteristics are presented in Table 3.

**Table 3:** Distribution of baseline ulcer characteristics across the three study groups The Pearson chi-square test was used for categorical variables, and one-way ANOVA was used for continuous variables; p < 0.05 was considered statistically significant.

Ulcer Characteristics	Group A (n=21)	Group B (n=21)	Group C (n=21)	Total (n=63)	χ² value	F value	p-Value
Laterality: right, n (%)	11	9	11	31	0.508	-	0.7757
Laterality: left, n (%)	10	12	10	32	-	-	-
Site: medial, n (%)	18	15	16	49	1.286	-	0.5258
Site: lateral, n (%)	3	6	5	14	-	-	-
Single ulcer, n (%)	19	18	20	57	1.105	-	0.5754
Multiple ulcers, n (%)	2	3	1	6	-	-	-
Recurrent ulcer, n (%) (Yes)	7	7	8	22	0.14	-	0.9325
Ulcer duration (weeks), Mean ± SD	30.62 ± 12.77	26.86 ± 14.75	23.43 ± 12.88	-	-	1.491	0.2334
Baseline ulcer area (cm²), Mean ± SD	14.56 ± 6.47	16.53 ± 8.35	17.12 ± 7.30	-	-	0.687	0.5071

Venous duplex findings across the three groups are presented in Table 4.

**Table 4:** Distribution of venous duplex findings across the three study groups The Pearson chi-square test was used; p < 0.05 was considered statistically significant. GSV, great saphenous vein

Venous Duplex Finding	Group A (PRP Application) (n=21)	Group B (PRP Injection) (n=21)	Group C (Saline Dressing) (n=21)	Total (n=63)	χ² value	p-Value
Reflux type: GSV, n (%)	15	10	13	38	5.105	0.2767
Reflux type: perforator, n (%)	6	7	6	19	-	-
Reflux type: mixed, n (%)	0	4	2	6	-	-

In both group A and group B, 16 out of 21 patients received four sessions of PRP, while the remaining five received three sessions. The mean number of PRP sessions was identical in both groups (3.76 ± 0.44), with no statistically significant difference (p = 1.0000) (independent t-test [mean sessions]: t = 0.000, p = 1.0000).

At six weeks, complete ulcer healing was observed in two (9.5%), seven (33.3%), and zero patients in groups A, B, and C, respectively. The difference in healing rates at six weeks was statistically significant across the three groups (p = 0.0064). Group B (PRP injection) demonstrated the highest healing rate at this early time point, whereas no patient in group C (saline dressing) achieved complete healing by six weeks. At three months, healing was achieved in eight (38.1%), eight (38.1%), and four (19.0%) patients in groups A, B, and C, respectively, with no statistically significant difference (p = 0.3097). At six months, 14 (66.7%), 15 (71.4%), and 9 (42.9%) patients achieved complete healing in groups A, B, and C, respectively. Although group C showed a lower healing rate, the difference did not reach statistical significance at the six-month endpoint (p = 0.1280). Complete ulcer healing at each follow-up time point is summarized in Table 5.

**Table 5:** Comparison of complete ulcer healing rates at six weeks, three months, and six months across the three study groups Chi-square test: χ² = 10.111, p = 0.0064

Time Point	Outcome	Group A (PRP Application) (n=21)	Group B (PRP Injection) (n=21)	Group C (Saline Dressing) (n=21)	Total (n=63)	p-Value
6 weeks	Healed	2 (9.5%)	7 (33.3%)	0 (0.0%)	9 (14.3%)	0.0064
	Not healed	19 (90.5%)	14 (66.7%)	21 (100.0%)	54 (85.7%)	
3 months	Healed	8 (38.1%)	8 (38.1%)	4 (19.0%)	20 (31.7%)	0.3097
	Not healed	13 (61.9%)	13 (61.9%)	17 (81.0%)	43 (68.3%)	
6 months	Healed	14 (66.7%)	15 (71.4%)	9 (42.9%)	38 (60.3%)	0.128
	Not healed	7 (33.3%)	6 (28.6%)	12 (57.1%)	25 (39.7%)	

Baseline ulcer areas were comparable across all three groups (p = 0.5071). At three months, significant differences emerged (p = 0.000039), with group B showing the lowest mean area (1.30 ± 1.61 cm²), followed by group A (3.15 ± 3.01 cm²) and group C (6.19 ± 4.49 cm²). At six months, groups A and B achieved near-complete reduction, whereas group C retained a mean area of 2.80 ± 3.03 cm² (p = 0.000035). Pairwise comparisons confirmed statistically significant differences between group C and the PRP groups at both three and six months. Serial changes in ulcer surface area are detailed in Table 6.

**Table 6:** Comparison of mean ulcer surface area at baseline, three months, and six months across the three study groups *One-way ANOVA was used for between-group comparisons at each time point. **Mean ulcer surface area was comparable among the three groups at baseline (p=0.5071), but was significantly lower in the PRP groups compared with the saline group at three and six months (p<0.0001), with a significant reduction in ulcer area over time within all three groups (p<0.0001). †Within-group p-values were obtained using repeated-measures ANOVA. A p-value of <0.05 was considered statistically significant.

Time Point	Group A (PRP Application), Mean ± SD (cm²)	Group B (PRP Injection), Mean ± SD (cm²)	Group C (Saline Dressing), Mean ± SD (cm²)	F value	p-Value*
Baseline	14.56 ± 6.47	16.53 ± 8.35	17.12 ± 7.30	0.687	0.5071
3 months	3.15 ± 3.01	1.30 ± 1.61	6.19 ± 4.49	12.072**	<0.0001**
6 months	0.33 ± 0.52	0.40 ± 0.88	2.80 ± 3.03	12.184**	<0.0001**
Within-group p-value†	<0.0001	<0.0001	<0.0001	-	-

At three months, group B demonstrated the highest mean percentage area reduction (91.80 ± 8.05%), followed by group A (78.29 ± 17.68%) and group C (62.34 ± 19.06%). The difference was statistically significant (p = 0.000001), with all pairwise comparisons showing significant differences. At six months, groups A and B achieved near-equivalent reductions (97.37% and 97.54%, respectively), while group C showed a notably lower reduction of 82.07 ± 16.02%, and this difference remained statistically significant (p = 0.000001).

Ulcer recurrence within six months was observed in one patient each in groups A and C and none in group B. The difference in recurrence rates was not statistically significant (p = 0.5967). Treatment-related complications were minimal: one patient in group A and two in group B reported pain or soreness, while one patient in group C developed superficial ulceration. The overall complication rate did not differ significantly across groups (p = 0.7657), indicating a comparable safety profile. The primary and secondary clinical outcomes are summarized in Table 7.

**Table 7:** Comparison of primary and secondary clinical outcomes among patients treated with PRP application, PRP injection, and saline dressing Pearson chi-square test was used for categorical variables, and one-way ANOVA was used for continuous variables. Mean healing-time ANOVA included only patients who achieved complete healing. A p-value of <0.05 was considered statistically significant. *Ulcer area reduction was significantly greater in the PRP-treated groups compared with the saline group overall. This study shows that PRP injections are not superior to PRP topical application, but can be a useful adjunct in early healing of chronic venous leg ulcers PRP, platelet-rich plasma; SD, standard deviation

Outcome Measure	Group A (PRP Application)	Group B (PRP Injection)	Group C (Saline Dressing)	χ² value	F value	p-Value
Complete healing at 6 weeks, n (%)	2 (9.5%)	7 (33.3%)	0 (0.0%)	10.111	-	0.0064
Complete healing at 3 months, n (%)	8 (38.1%)	8 (38.1%)	4 (19.0%)	2.344	-	0.3097
Complete healing at 6 months, n (%)	14 (66.7%)	15 (71.4%)	9 (42.9%)	4.112	-	0.128
Percentage area reduction at 3 months, mean ± SD (%)	78.29 ± 17.68	91.80 ± 8.05	62.34 ± 19.06	-	18.498	<0.0001*
Percentage area reduction at 6 months, mean ± SD (%)	97.37 ± 3.96	97.54 ± 4.03	82.07 ± 16.02	-	17.227	<0.0001*
Mean healing time (weeks), mean ± SD	16.07 ± 3.87	11.20 ± 2.18	17.67 ± 4.24	-	12.455	0.0001*
Recurrence at 6 months, n (%)	1 (4.8%)	0 (0.0%)	1 (4.8%)	1.033	-	0.5967
Any complication, n (%)	1 (4.8%)	2 (9.5%)	1 (4.8%)	0.534	-	0.7657

Group B (PRP injection) demonstrated the earliest healing and the highest percentage area reduction at three months. At six months, groups A and B showed comparable and near-complete area reduction, both significantly superior to group C. Healing time was significantly shorter in group B compared to groups A and C. Recurrence and complication rates were comparable across all three groups, indicating equivalent safety profiles.

## Discussion

In this study, all three groups were comparable at baseline with respect to age, sex, BMI, glycemic status, platelet count, ABPI, ulcer duration, ulcer size, laterality, recurrence status, and other ulcer characteristics, supporting the validity of outcome comparisons. The major finding was that PRP injection (group B) demonstrated superior early healing, with the highest complete healing rate at six weeks, greater reduction in ulcer area at three months, and the shortest healing duration. PRP application (group A) also showed better outcomes than saline dressing but with a slower initial response. Recurrence and treatment-related complications remained low and comparable among all groups.

The beneficial effects of PRP are explained by its high concentration of platelet-derived growth factors and cytokines, which enhance angiogenesis, fibroblast proliferation, granulation tissue formation, and epithelial regeneration in chronic wounds [15,16]. The demographic profile and ulcer characteristics in our study were comparable with previous studies by Elbarbary et al. and Moneib et al., which evaluated similar populations with CVLUs [14,17].

PRP injection resulted in significantly better early complete healing at six weeks, with healing achieved in 33.3% of patients compared with 9.5% in the PRP application group and none in the saline group. These findings are consistent with Elbarbary et al., who reported superior healing outcomes with local PRP injection compared with PRP dressing and compression therapy alone [14]. The improved response may be due to direct delivery of growth factors into the ulcer margins and deeper viable tissues, promoting angiogenesis and tissue repair more effectively [15].

Although complete healing rates at three and six months did not reach statistical significance, the PRP groups consistently showed better outcomes, which may be related to the limited sample size. Meta-analysis by Fang et al. demonstrated improved healing rates and reduced ulcer area with PRP compared with conventional treatment [18]. Similarly, Yammine et al. reported enhanced healing outcomes with PRP without increased infection risk [15].

In our study, ulcer area reduction was significantly greater in both PRP groups, with PRP injection showing the highest improvement at three months. PRP injection also significantly shortened the healing time compared with PRP application and saline dressing. The favorable safety profile, with minimal adverse effects and low recurrence, supports the use of PRP as an effective adjunct to compression therapy, with injection delivery providing the greatest potential for accelerated healing in CVLUs.

Recommendations

Our investigation revealed that autologous PRP, especially when delivered via injection, improves healing in CVLUs relative to saline dressing. PRP injection demonstrated the most favorable early healing response, the highest percentage decrease in ulcer area at three months, and the shortest healing duration, while both PRP methods attained almost full area reduction by six months and sustained low rates of recurrence and complications.

The findings are robustly corroborated by other published research, particularly the randomized trial conducted by Elbarbary et al., alongside other prospective studies and recent meta-analyses demonstrating superior healing outcomes with PRP compared to usual therapy. While earlier evaluations warned of inadequate evidence quality, newer research increasingly endorses PRP as a safe and effective supplement in the therapy of VLU. Collectively, our findings indicate that PRP injection may represent the most efficacious method of autologous PRP administration for expediting healing in CVLUs.

Limitations

Notwithstanding the promising results, our work must be considered in the context of several limitations. The limited sample size, single-center design, and limited blinding possibly diminish the capacity to identify statistically significant differences in full healing rates at three and six months despite the evident numerical advantage of the PRP groups. Baseline imbalances were seen in smoking history, prior DVT, and frequency of debridement. While these factors do not undermine the robust continuous-outcome data, they may have affected the healing dynamics to a certain degree.

Also, while enrolling participants into the study, only the chronicity of VLU was noted. There was no consideration given to the CEAP (Clinical Etiological Anatomical Pathophysiological) classification of each participant, which could have given insight into the severity of the underlying chronic venous disease. The severity of reflux in the superficial/deep venous system along with that in the perforators has an inverse relation to the healing of VLU, which was also not considered in this study. An understanding of severity of the underlying pathology could have influenced the frequency of debridement in the participants across all 3 groups, but this study could not provide the relevant statistics for the same.

These limitations parallel issues identified in previous systematic studies, which highlighted variability in PRP preparation techniques, treatment protocols, compression application, wound length, and outcome criteria. Although our study provides significant data supporting PRP injection, particularly for the acceleration of early healing, larger multicenter randomized studies with standardized PRP procedures and extended follow-up are necessary to formulate definitive treatment guidelines.

## Conclusions

Our investigation revealed that autologous PRP, especially when delivered via injection, improves the initial healing in CVLUs relative to saline dressing. PRP injection demonstrated the early healing response with the highest percentage decrease in ulcer area at three months and the shortest healing duration, while both PRP methods attained almost full area reduction by six months and sustained low rates of recurrence and complications.

While earlier evaluations warned of inadequate evidence quality, newer research increasingly endorses PRP as a safe and effective supplement in the therapy of VLU. Collectively, our findings indicate that PRP injection may represent the most efficacious method of autologous PRP administration for expediting healing in CVLUs.
